# Medical residents’ burnout levels, correlates and personal attributions: a pilot study to inform the development of a digital intervention tool

**DOI:** 10.1192/j.eurpsy.2026.12059

**Published:** 2026-07-31

**Authors:** A. T. Pereira, M. J. Brito, A. I. Araújo, C. Cabaços, J. S. Silva, A. Macedo

**Affiliations:** 1 Psychological Medicine, Faculty of Medicine University of Coimbra; 2 Electrical and Computer Engineering, University of Coimbra, Coimbra, Portugal

## Abstract

**Introduction:**

Burnout is highly prevalent in medical career, peaking in residency. Our research with medicine students revealed that their high perfectionism and related emotional regulation strategies are risk factors for burnout.

**Objectives:**

This pilot study is part of a new project “RESOUT - Smartphone app for RESident physicians’ burnOUT monitoring and prevention” (FCT 2023.13877.PEX) and aims to explore levels of residents’ burnout and to identify its individual and contextual correlates, in order to empirically inform the assessment and intervention strategies to be included in the app and the secondary outcomes to considered when analysing its effectiveness.

**Methods:**

To date, 300 Pt residents (75% women) completed questionnaires to evaluate Burnout, Psychological distress/PD (depression/anxiety/stress) symptoms and attributions, Perfectionism, Worry and rumination/WR, Self-compassion/SC, Perceived Life Satisfaction/PLS and Occupational Satisfaction/POS, and Perceived-self-efficacy/PSE, -empathy/PE and -medical errors/PME.

**Results:**

More than half of the sample had moderate (35.2%) / high (21.6%) burnout levels. Among these, 38.9% report thinking about leaving residency often/very, being the most mentioned reasons to do it: high workload (72%), lack of personal/family time (73.6%) and rewards (51%) and difficulty dealing with stress (48.3%).

Perfectionism, WR and low SC were predictors of burnout (betas>.40). SC mediates the relationship between the other predictors and burnout. Both perfectionism and SC moderate the relationship between have mentioned those reasons and burnout.

Burnout (or at least one of its dimensions) predicted low PLS (-.589), low PRS (-.524), PD (.677), frequency of leaving residency thoughts (.583), low PSE (-.324), PE (.665) and PME (.224) (all *p≤.001*).

**Conclusions:**

These findings confirm that provide residents with SC practices may help them manage perfectionism and reduce burnout symptoms and its negative impact on them and their patients. Considering they attribute their distress to the high job demands they face, digital interventions – accessible, flexible, AI-powered personalized - can promote their adherence.

**Disclosure of Interest:**

None Declared

